# X-ray microscopy enables multiscale high-resolution 3D imaging of plant cells, tissues, and organs

**DOI:** 10.1093/plphys/kiab405

**Published:** 2021-09-27

**Authors:** Keith E Duncan, Kirk J Czymmek, Ni Jiang, August C Thies, Christopher N Topp

**Affiliations:** Donald Danforth Plant Science Center, St Louis, Missouri 63132, USA

## Abstract

Capturing complete internal anatomies of plant organs and tissues within their relevant morphological context remains a key challenge in plant science. While plant growth and development are inherently multiscale, conventional light, fluorescence, and electron microscopy platforms are typically limited to imaging of plant microstructure from small flat samples that lack a direct spatial context to, and represent only a small portion of, the relevant plant macrostructures. We demonstrate technical advances with a lab-based X-ray microscope (XRM) that bridge the imaging gap by providing multiscale high-resolution three-dimensional (3D) volumes of intact plant samples from the cell to the whole plant level. Serial imaging of a single sample is shown to provide sub-micron 3D volumes co-registered with lower magnification scans for explicit contextual reference. High-quality 3D volume data from our enhanced methods facilitate sophisticated and effective computational segmentation. Advances in sample preparation make multimodal correlative imaging workflows possible, where a single resin-embedded plant sample is scanned via XRM to generate a 3D cell-level map, and then used to identify and zoom in on sub-cellular regions of interest for high-resolution scanning electron microscopy. In total, we present the methodologies for use of XRM in the multiscale and multimodal analysis of 3D plant features using numerous economically and scientifically important plant systems.

## Teaser

Lab-based X-ray microscopy allows high-resolution 3D imaging of intact plant samples over a wide range of sample types and sizes, filling the imaging gap between light and electron microscopy.

## Introduction

Visible features of the plant, such as flowers, leaves, and roots, result from microscopic processes of tissue and organ formation, which are in turn driven by cellular and molecular dynamics. How whole plants and their organs, tissues, cells, organelles, and sub-cellular molecules interact within three-dimensional (3D) space is crucial to understanding plant biology across diverse genetic backgrounds and changing environments. Capturing 3D external and internal volume data brings an important spatial component to plant phenotype analysis that can help identify genetic factors that control trait development not manifested in simpler 2D images. Recently, 3D imaging platforms and corresponding phenotyping methods have developed rapidly, and many approaches have been applied to a wide diversity of plant growth and development phenomena (Morris et al., 2017; Perez-Sanz et al., 2017; Tracy et al., 2017; Jiang et al., 2019; Roth et al., 2019; Strock et al., 2019; Czymmek et al., 2020; Li et al., 2020). Furthermore, sophisticated analysis pipelines have been developed to extract and quantify data from relevant features, with improved imaging leading directly to improved trait analysis and models (Bassel and Smith, 2016; Koebernick et al., 2017; Mathers et al., 2018; Jackson et al., 2019; McKay Fletcher et al., 2020; Sultan et al., 2020; Théroux-Rancourt et al., 2020; Wolny et al., 2020).

Current 3D systems use photons, electrons, and X-rays for imaging biological samples. Photon-based tomography (confocal, multiphoton, light-sheet, super-resolution) generates valuable 3D information with chemical specificity based on fluorescent probes and/or auto-fluorescence in both living and fixed samples (Wymer et al., 1999; Feijó and Moreno, 2004; Schubert, 2017; Ovečka et al., 2018). However, imaging with photons fundamentally has sample size constraints (Prunet and Duncan, 2020; Rawson et al., 2020) and nondestructive approaches (but see (Strock et al., 2019) are typically limited to shallow image depths for plant tissues as they are highly refractive due to cell walls, vacuoles, and air spaces, that often require special preparation or clearing to mitigate (Littlejohn et al., 2010; Kurihara et al., 2015). Another approach, 3D volume scanning electron microscopy (SEM), repeatedly images a resin block-face after removing thin layers via an *in situ* microtome or surface ablation via focused ion beam (Bhawana and Cahoon, 2014; Kittelmann et al., 2016; Czymmek et al., 2020; Harwood et al., 2020). With volume SEM, detailed 3D spatial information is possible down to a few nanometers; however, practical imaging volumes are typically restricted to a maximum of a few hundred micrometers (Kubota et al., 2018). Additionally, sample preparation is highly specialized, where prolonged protocols with greatly increased sample staining are required to ensure adequate signal-to-noise and sample resolution.

The X-ray microscope (XRM) is a specialized X-ray computed tomography (CT) instrument that incorporates microscope objective lenses (Jacobsen, 2020). In XRM, 2D digital radiographs are projected onto a scintillator-coated objective lens where the X-ray signal is converted into visible light, magnified, and the resulting image collected with a detector, typically a CCD camera. The objective lens in the beam path increases the resolution of the XRM compared with conventional X-ray CT systems, which rely solely on geometric magnification, and multiple objective lenses make possible the collection of 3D volumes over a wide range of sample sizes and resolutions not practical with other imaging platforms. Furthermore, in contrast to photon- and electron-based tomography, where imaging penetration depths are restricted to one or a few cell layers, the ability of X-rays to penetrate through large, intact and highly light scattering plant structures is a distinct advantage. Thus, lab-based XRM provides a critical link between photon and electron 3D imaging, establishing high-resolution intermediate magnification levels for whole plant imaging.

X-ray imaging contrast is based on the differential density of features within a specimen; however, most biological samples are composed of relatively uniform low-density organic material, often with insufficient contrast to generate useful 3D image data. For this reason, sample fixation protocols and contrast agents play important roles in imaging biological samples with XRM (Metscher, 2009; Rawson et al., 2020). Staedler et al. (2013) evaluated conventional EM sample fixation methods and contrast agents as tools for examining plant samples with XRM. These methods were sufficient for the relatively short scan times necessary to capture floral anatomy for analysis using basic morphological markers, but do not provide adequate stability for the longer scan times required for high magnification/resolution of more subtle and complicated plant features.

Here, we have built upon these earlier methods, incorporated improvements in sample preparation, and leveraged instrument technological advances to expand the range of plant cells, tissues, and organs that can be imaged at high resolution with XRM. This enabled unprecedented 3D perspectives and insights for phenotype and form-function studies. Further, due to the improved image quality of 3D volume data, advanced image analysis and cell-level segmentation of relevant structures of interest using computer vision and learning are substantially enhanced. We applied XRM with improved sample preparation for multimodal and multiscale 3D imaging, where samples prepared for high-resolution volume SEM were first imaged with XRM as part of a correlative workflow (Bushong et al., 2015; Tsang et al., 2018; Mitchell et al., 2019; Bayguinov et al., 2020). Taken together, we anticipate that the adoption of these improved techniques will make an important contribution to plant biology, expanding the reach of XRM as a routine tool for 3D imaging for plant scientists.

## Results

### Meristem biology

**Figure 1:**
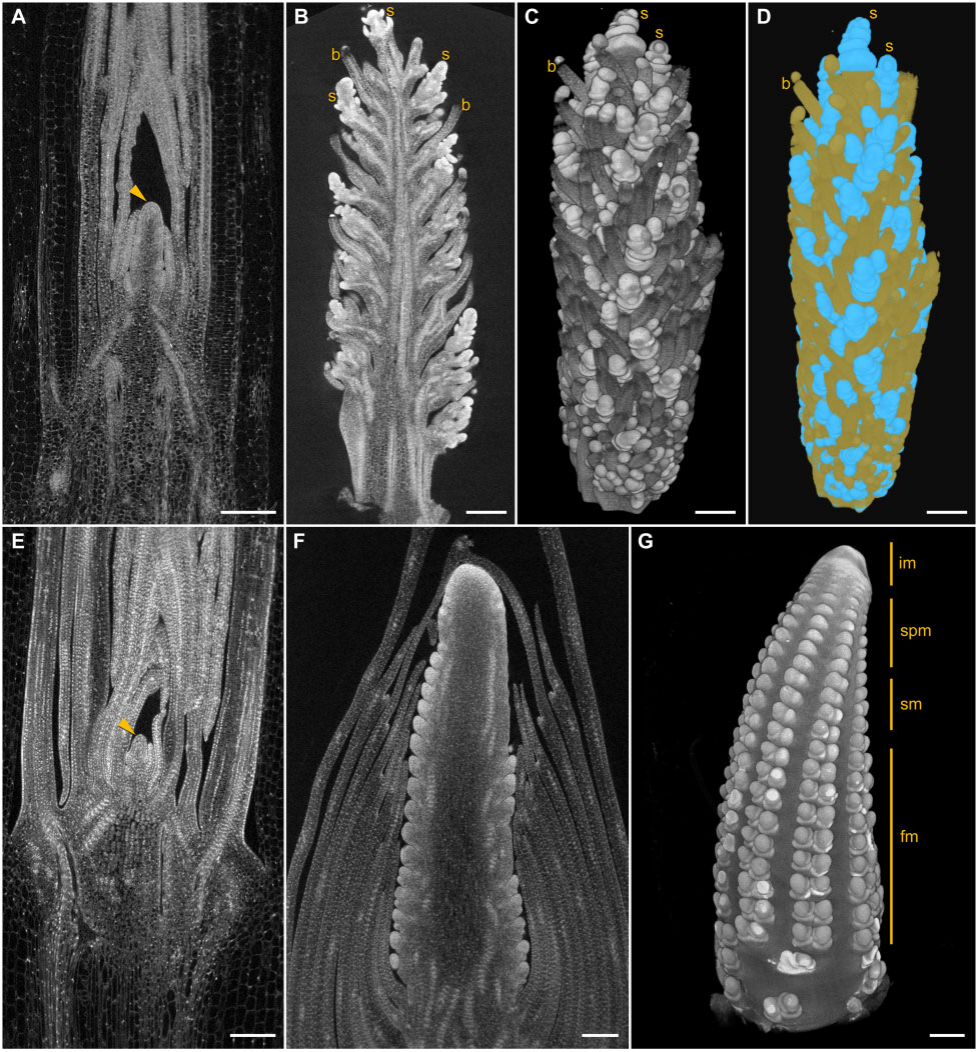
Meristem biology. Shoot apical meristems (arrowheads) from *Setaria viridis* (A) and *Zea mays* (E) illustrate how the development of this important organ can be visualized in 3D at high-resolution while remaining intact within surrounding sheath tissue. Inflorescence structure from *S. viridis* was scanned (B), displayed as a volume rendering (C), and segmented (D) to identify individual spikelets (s) and bristles (b). X-ray microscope imaging of *Z. mays* ear primordium (F), with the volume rendering (G) providing high-resolution 3D details of regions containing developing inflorescence (im), spikelet pair (spm), spikelet (sm), and floral meristems (fm). All scale bars 200 µm.

Our ultimate goal was to develop plant-specific XRM protocols and workflows that improved specimen contrast with excellent stability for long-duration high-resolution scans^1^. To image plant tissue with XRM, differential contrast of cell walls was particularly useful, for example, in visualizing individual cells found in root tip samples that had large central vacuoles and little cytoplasm; however, this is difficult to achieve in cytoplasm-rich meristematic tissue. For wet preparations, we routinely achieved high contrast using ethanolic phosphotungstic acid (ePTA) and improved sample stability by mounting specimens in low melting point (LMP) agarose (see Supplemental Figure S1). Longer scans were made possible by improved sample contrast and stability, producing better signal-to-noise ratios and edge sharpness in images, both of which helped differentiate individual meristematic tissues within inflorescence structures. Figure 1 illustrates how our XRM approach addressed this challenge with examples of meristematic tissue from *Setaria viridis* and maize (*Zea mays*). Figure 1, A and E show ePTA contrasted shoot apical meristems from *S. viridis* and maize, respectively. These methods were also effective for generating detailed 3D volumes of inflorescence structures that allowed visualization of spikelets and sterile bristles in *S. viridis* (Figure 1, B and C) where the inherent difference in density between tissues was observed as variations in grayscale, illustrated in the volume segmentation in (Figure 1, D;Supplemental Figure S2). Similar imaging detail was achieved in ear primordia of maize (Figure 1, F and G) where inflorescence, spikelet pair, spikelet, and floral meristem features were well-contrasted and resolved for 3D measurement.^1^

### Floral anatomy and development

**Figure 2:**
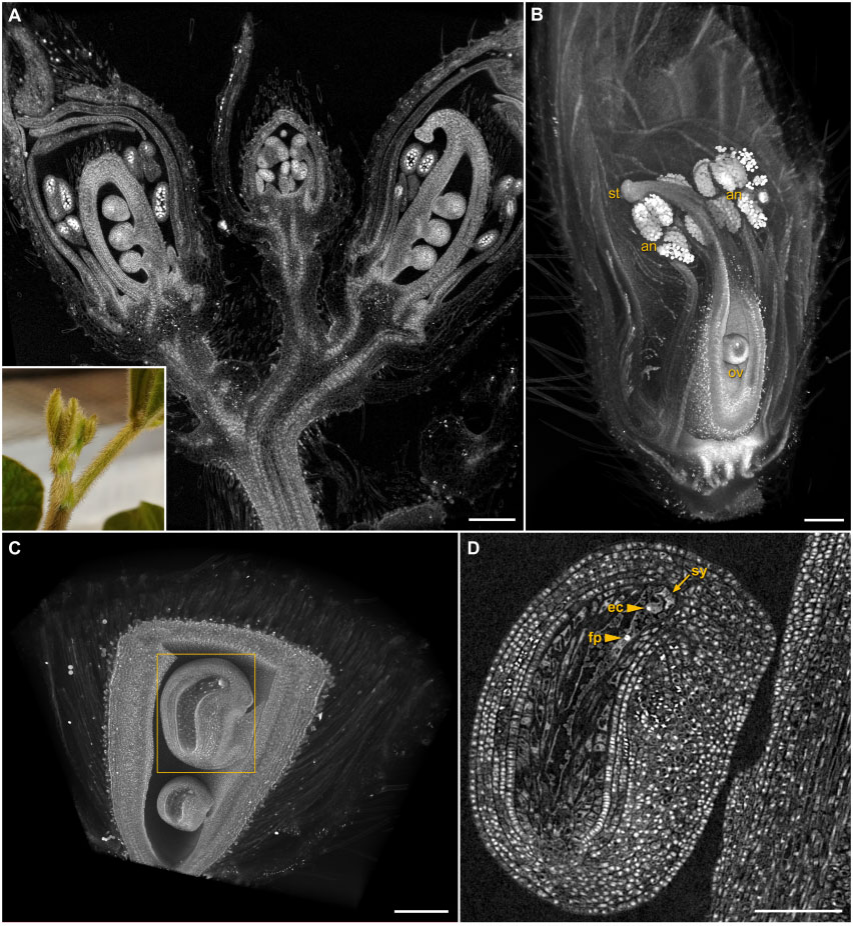
Multiscale imaging of floral anatomy and development. Fixed and contrast-enhanced reproductive structures of *Glycine max*. A, Axillary buds (A, inset) contrasted in phosphotungstic acid, the entire intact sample imaged with XRM for a detailed 3D volume of this complicated structure; scale bar 300 µm. B, Volume rendering of a single developing flower shows the relationship of pollen-filled anthers (an), ovules (ov), and stigmatic surface (st) to one another in 3D space; scale bar 200 µm. C, Volume rendering of developing ovules; scale bar 200 µm. D, High magnification scan of indicated ovule from (C) illustrates cell-level resolution of egg cell (ec) and fused polar (fp) nuclei, synergids (sy); scale bar 100 µm.

Next, we applied this approach to soybean (*Glycine max*), whose indeterminate growth regularly produces flowers at multiple developmental stages. The relatively large (∼1 cm) axillary bud complex (Figure 2A inset) was difficult to image as a single unit in 3D with other microscopic technologies, but was imaged in its entirety with XRM as shown in Figure 2A. Reproductive structures including anthers, pollen, ovules, and the stigmatic surfaces from multiple developmental stages can be seen in high detail (Figure 2A). A higher-resolution scan of a single developing flower (Figure 2B) shows the 3D spatial relationships of pollen-filled anthers to the stigma, stigmatic surface, and ovules. Paired ovule scans demonstrate the multiscale capability of XRM (Figure 2, C and D). An overview scan at 1.5-µm voxel resolution of two pollinated ovules (Figure 2C) was used to select a specific ovule-of-interest for a higher-resolution scan at 0.6-µm voxel resolution (Figure 2D), where details like the egg cell nucleus, fused polar nuclei, and developing synergids were evident. This example clearly shows the effectiveness of these methods for generating high-resolution scans over different scales from a single mounted specimen. Additional examples of the benefits of multiscale imaging of a single mounted sample with XRM include *S. viridis* and *Eragrostis tef* (Supplemental Figure S3) and *Thlaspi arvense* (Supplemental Figure S4, A and B), where multiscale 3D imaging captured entire intact inflorescence structures, followed by micrometer scale imaging of internal features such as cotyledons, ovaries, ovules, stigmas, and anthers. Multiscale imaging of Arabidopsis *(Arabidopsis thaliana)* plantlets also demonstrated the suitability for visualization of the shoot apical meristem in agarose-stabilized samples (Supplemental Figure S4, C and D).

### Root biology

**Figure 3:**
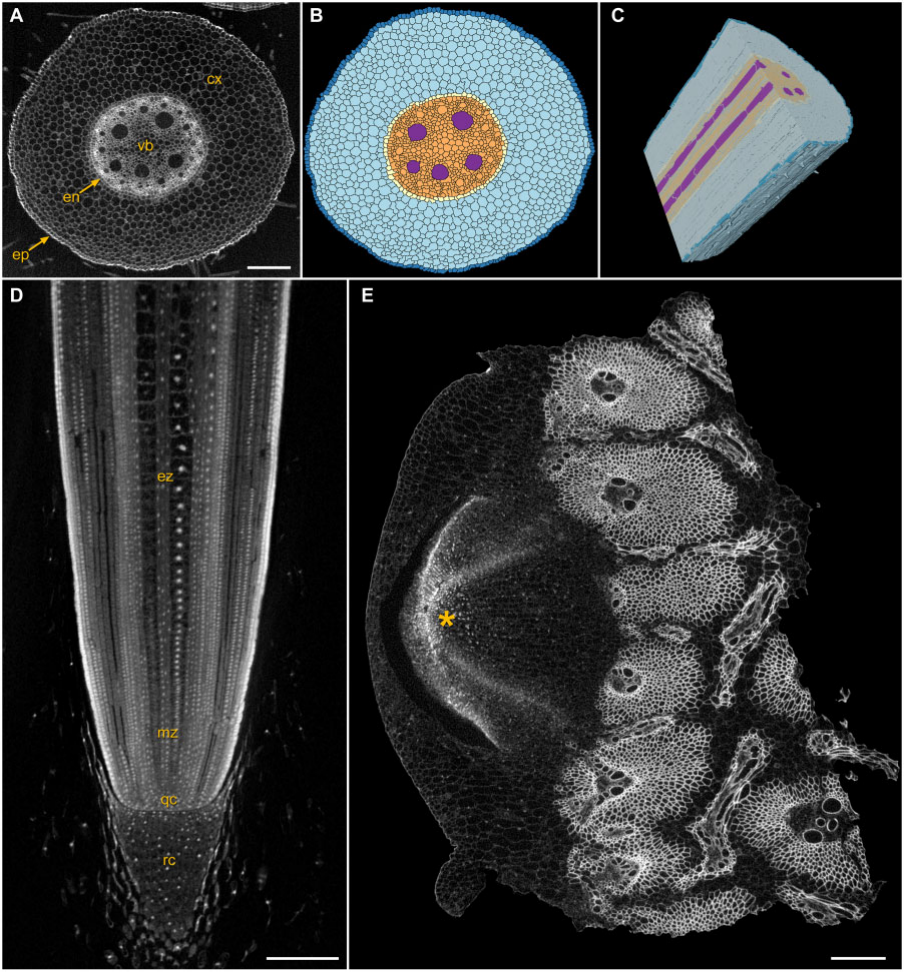
Root biology. A–C, *Zea mays* root tip fixed and contrast enhanced to visualize distinct cell layers: epidermis (ep), cortex (cx), endodermis (en), vascular bundle (vb), with computational segmentation at both the cell (B) and tissue (C) levels; scale bar 200 µm. D, Longitudinal view through the root tip where nuclei are easily visualized in the root cap (rc), quiescent center (qc), and meristematic (mz) and elongation (ez) zones; scale bar 200 µm. E, Stalk sample from the second above-ground node of a *Z. mays* plant at V8, contrasted in Lugol’s iodine, showing a brace root primordium (asterisk) and the well-contrasted vascular bundles; scale bar 300 µm.

The extension and curvature of roots in 3D space are driven by cell division and elongation patterns, but the roots of most plants are too thick for light microscopy to visualize internal cell layers from intact samples that are critical to these dynamics. XRM scanning of fixed and contrast-enhanced maize intact root tips (Figure 3, A and D) allowed 3D visualization and computational segmentation at both the cell (Figure 3B) and tissue level (Figure 3C), with cell volume data presented in Supplemental Figures S5 and S6. Here, root tips were contrasted with either aldehyde-osmium protocol (Figure 3A) or with ePTA (Figure 3D), and stabilized in agarose for the long scan times required to visualize individual cell walls and nuclei throughout the full 3D volume (Figure 3D). Root tip morphology was compared with published research for identification of specific cell types (Saleem et al., 2010; Alarcon et al., 2014), allowing clear visualization of the epidermis, cortex, endodermis, and stele tissues. In a thick sample of a maize node (5 mm), vascular tissues showed excellent contrast with iodine-based treatment (Figure 3E) and the complicated architecture of xylem, phloem, and brace root primordia at the nodal plexus were readily observed.

### Plant–microbe interactions

**Figure 4:**
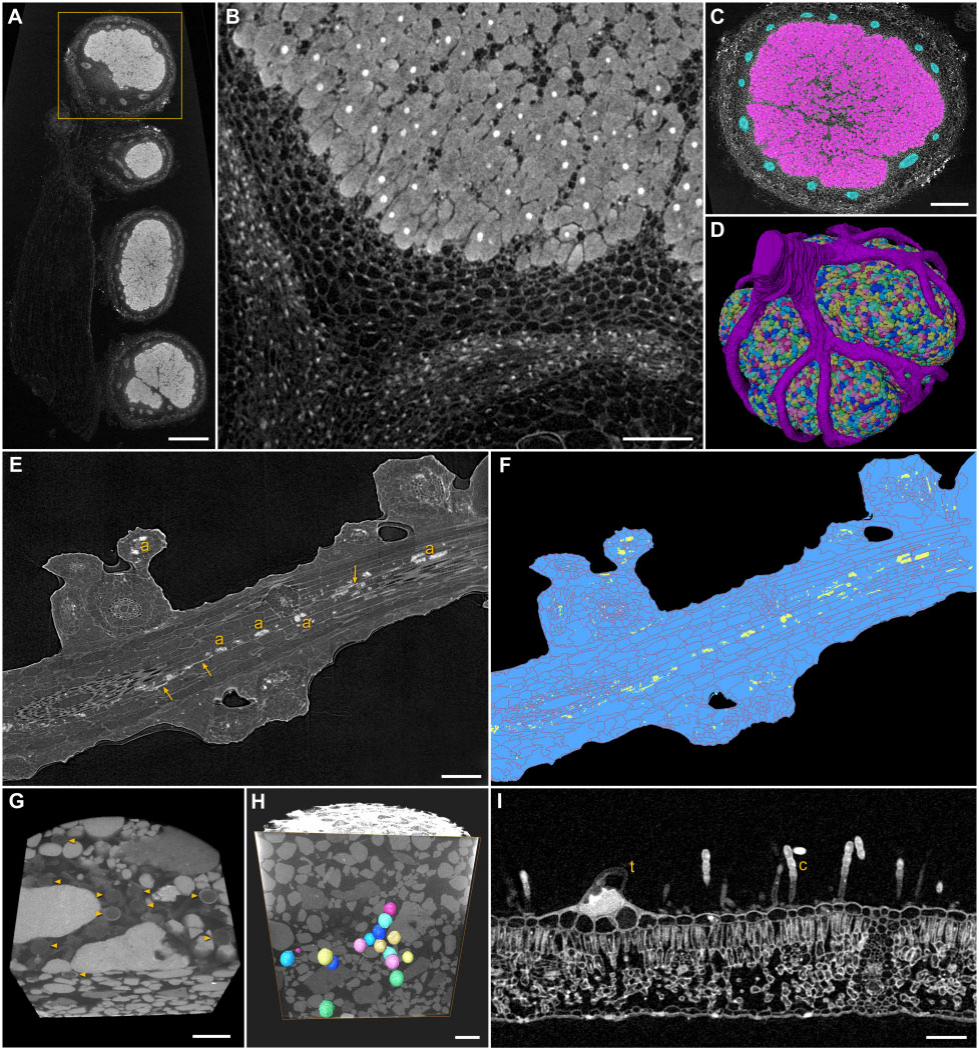
Plant–microbe interactions. A, Low magnification scan of *Bradyrhizobium japonicum* nodules on soybean root; scale bar 500 µm. B–D, High-resolution scan and segmentation of nodule from (A). Well-contrasted nuclei and cell walls allow segmentation of symbiosomes and vasculature; scale bars (B) 150 µm, (C) 350 µm. E and F, Colonization of maize root by *Rhizophagus irregularis*, arbuscules (a) and intercellular hyphae (arrows) segmented in yellow; scale bar 200 µm. G and H, Volume rendering of *Gigaspora margarita* spores *in situ* (arrowheads, G), spores segmented in color (H); scale bars 500 µm. I, Trichome (t) and fungal conidiophores with conidia (c) on *Echinacea* leaf surface; scale bar 150 µm.

3D visualization of plant–microbe relationships *in situ* and across scales can provide valuable data about how organisms interact in response to various environmental stimuli and genetic backgrounds. For example, nitrogen-fixing bacteria colonize the roots of many leguminous crops, providing host-accessible forms of nitrogen to the plant in exchange for carbon-based energy and a protected environment (Coba de la Peña et al., 2017). Here, *Bradyrhizobium japonicum* was applied to wild-type soybean seedlings, and the resulting nodules were harvested after 14 d. The Zeiss Xradia 520 Versa XRM has a stitching function that allows multiple overlapping high-resolution scans to be collected over a large region of a single sample, which are computationally assembled into a single 3D volume by the Zeiss Reconstructor software. In Figure 4A, a root segment with four nodules was imaged with a two-panel stitched XRM scan, and a subsequent higher-resolution scan in a selected region of the nodule (Figure 4B) showed well-contrasted plant cells and their nuclei, densely packed with symbiosomes and the surrounding nodule vascular network. The strong affinity of PTA for nuclei (Hayat, 1993) assisted with segmentation, providing a consistent central feature to guide the identification of individual cells, combined with the sharp edge contrast of the surrounding plant cell walls allowed detailed segmentation via a combination of manual and automatic approaches (Figure 4, C and D).

Mycorrhizal fungi colonize root cortical cells from most plant families, providing water and nutrients to the host in exchange for sugars and fatty acids (Luginbuehl and Oldroyd, 2017; Chen et al., 2018). To test the suitability of our XRM methods for mycorrhizal associations, wild-type maize plants were grown in the greenhouse in a mixture of field soil and sand, inoculated with spores of the arbuscular mycorrhizal fungus *Rhizophagus irregularis*, and colonized roots were harvested after 6 weeks. These roots were fixed and contrast-enhanced with ePTA and imaged. As Figure 4E illustrates, arbuscules—the symbiotic structures formed within the cortical cells to exchange nutrients between host plant and fungus—were clearly visible within maize cortical cells. Intercellular hyphae were also evident by segmentation of these structures shown in Figure 4F. In an alternative system, we grew *Medicago sativa* in 20-mL syringe barrels with a sand-Agsorb^®^ mixture inoculated with spores of *Gigaspora margarita.* To image both symbiotic partners over scales, we first used the XRM flat panel detector which allowed us to visualize the entire root system in a 10 cm × 2 cm syringe barrel. We then scanned specific regions of interest within the barrel (using the 0.4× and 4× objective lenses), to localize *G. margarita* spores *in situ* (Figure 4, G and H; Supplemental Figure S7). This multiscale approach yielded unperturbed 3D volume data of fungal structures in relation to the entire host root system without removing the plant from the soil. To evaluate host–pathogen interactions, we imaged powdery mildew infected leaves of *Echinacea* spp. that were collected from a prairie grassland ecosystem, fixed in buffered aldehydes and contrast enhanced with osmium tetroxide, and imaged via XRM. Conidiophores with conidia emerged from the surface of the leaf in Figure 4I, adjacent to host trichomes.

### Correlative microscopy

**Figure 5:**
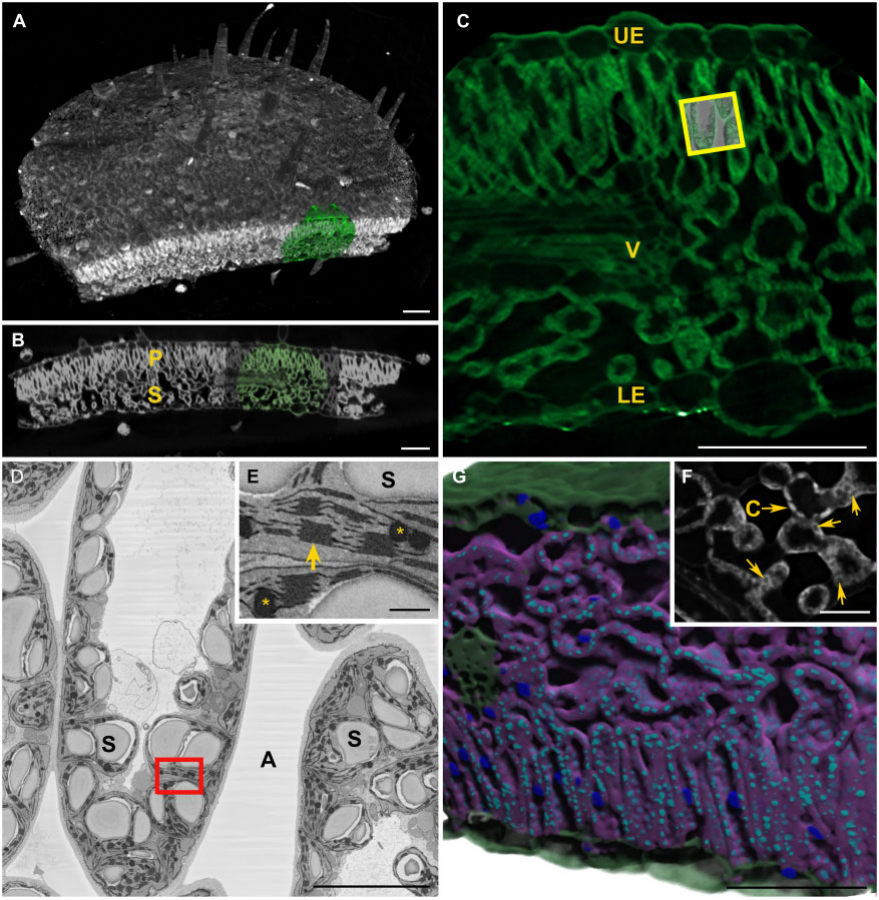
Multiscale correlative X-ray and electron microscopy of *N. benthamiana* leaf. Resin embedded *N. benthamiana* leaf prepared using OTO staining protocol provided high contrast 3D (A) and 2D (B) XRM perspectives for corresponding high-resolution XRM (C, A, and B, green). This approach provided 3D detail of the palisade (P) and spongy (S) mesophyll and cell wall defining the vascular tissue (v) and upper (ue) and lower (le) epidermis enabling rich content for subsequent correlative SBFEM (C, EM overlay, yellow box) at an intermediate resolution of palisade cells (D, corresponding to (C) yellow box) with a complement of cell organelles, including starch (s) and apoplast (a) and high-resolution inset (E, corresponding to (D) red box) with grana (arrow) and plastoglobules (asterisks). High-resolution XRM in spongy mesophyll (F) shows chloroplast (c) and starch voids (arrows). Deep learning of XRM data allowed 3D segmentation/visualization (G) of nuclei (dark blue), starch granules (light blue), cell cytoplasm/chloroplasts (purple), and cell walls (green). Scale bars 100 µm (A–C, and G), 10 µm (D), 500 nm (E), and 20 µm (F).

One challenge facing plant biologists applying high-resolution electron microscopy is targeting specific or rare structures within the plant and/or locating very small regions acquired by EM in the larger bulk plant tissue. This becomes especially problematic when dealing with opaque heavy metal stained and resin-embedded EM plant samples, where features are often not visible in the densely stained and osmicated tissue. Here we applied an enhanced staining approach used to achieve uniform staining for serial block face electron microscopy (SBFEM) in brain tissues (Hua et al., 2015) to *Nicotiana benthamiana* leaf tissue which had been embedded in epoxy resin. Our goal was to provide a high contrast/resolution protocol for tissue and subcellular details that was optimized for multi-scale correlative XRM/SBFEM. With this preparation, we generated an overview 3D XRM tomogram using a 4× objective lens at a 1.2-µm voxel size (Figure 5A). Under these conditions, the entire 3-mm diameter leaf punch could be readily interrogated and clearly revealed the spatial distribution of plant structures including trichomes, palisade mesophyll, spongy mesophyll, and vascular tissue at arbitrary slice perspectives (Figure 5B). Subsequently, an XRM scan using the 40× objective lens was applied at 0.2-µm voxel size for visualization of a high-resolution subset of the leaf that was submerged into the lower resolution dataset (Figure 5, A and B, green cylinder). A representative 2D slice of the *N. benthamiana* leaf cross-section within the high-resolution volume (Figure 5C) shows the spatial distribution and more detailed tissue features and enabled precise targeting of individual palisade cells for subsequent imaging (Figure 5C, yellow box, SBFEM overlay). We next targeted the palisade mesophyll for high-resolution SBFEM in the same specimen to directly correlate individual chloroplasts at nanometer scale within the 3D XRM volume (Figure 5, D and E). The enlarged single 2D slice (Figure 5D) corresponding to Figure 5C from a series of approximately 1,500 SBFEM images of a palisade cell showed multiscale image correlation and highlights the resolution gain from SBFEM for nanoscale features. Subcellular structures such as starch, thylakoid membranes, plastoglobules, and individual grana and stacking were readily documented. With the strong staining provided by this protocol combined with the stability afforded by epoxy resin embedding for long term high-resolution XRM imaging, we were able to delineate individual isolated chloroplasts, and sub-chloroplast voids corresponding to starch granules (Figure 5F), despite the fact that the cytoplasmic cell periphery was often densely packed, with contrast largely coming from intensely stained chloroplast membranes. Segmentation using machine learning of high-resolution leaf features permitted clear 3D visualization and distribution of nuclei, cell walls/vascular bundle, chloroplast distribution, and their starch granules across tissues (Figure 5G).

## Discussion

Rapid technological and computational improvements over the past several decades have enabled the acquisition and analysis of 3D volumetric data of ever-increasing size and complexity for plant biology. However, intact plant structures are often difficult to image in 3D with light and/or electron microscopy platforms due to challenges including relatively large sample size, an optically scattering and chemically resistant cuticle, cell walls and air pockets that impede fixation and exogenous treatments, complex morphologies, and delicate features. As a result, a notable gap exists with light and electron imaging modalities that restrict high-resolution tomographic imaging to small samples that provide limited contextual information relative to the whole organ or plant.

Because of its ability to penetrate dense tissues, conventional X-ray CT was utilized to address some of these imaging limitations in plant science as early as the 1990s (Aylmore, 1993; Heeraman et al., 1997) and 2000s (Stuppy et al., 2003; Kaminuma et al., 2008; Leroux et al., 2009; Dhondt et al., 2010; van der Niet et al., 2010). However, most conventional X-ray CT instruments rely solely on geometric magnification, and thus have functional resolution and sample size limits based on source-sample-detector geometry. Synchrotron-based X-ray CT has advantages in shorter scan times, high-resolution imaging, and allows element mapping using X-ray fluorescence (Kopittke et al., 2018); however, their limited availability relative to lab-based XRM restricts widespread utilization in plant biology. Here, we demonstrated that lab-based XRM could image intact plant structures in fine detail, and subsequently zoom to selected regions of interest down to the cellular and subcellular level, effectively bridging the tomographic imaging gap of light and electron microscopy.

Contrast and stabilization of samples are critical for high-resolution X-ray imaging due to the longer scan times. Previous XRM studies used conventional sample fixation, contrast enhancement, dehydration, and critical point drying followed in some cases by sputter coating (Staedler et al., 2013; Bellaire et al., 2014; Jeiter et al., 2018; Jeiter et al., 2020; Vasile et al., 2021). Other researchers stabilized samples in the contrast enhancement medium, sealed within pipette tips or polypropylene tubes (Gamisch et al., 2013; Staedler et al., 2013, 2018; Reich et al., 2020). In these studies, the goal was to survey a relatively large number of samples and evaluate basic 3D shapes using scans no longer than necessary to achieve stated research aims in a timely manner. With plant tissue prepared in this way, even with floral samples stabilized in polyester fibers or in ethanol, sample drift during longer XRM scans can be problematic and therefore limit achievable resolution and magnification. To address this challenge, we specifically developed a simple but effective strategy whereby fixed samples were contrasted in ePTA and stabilized in LMP agarose inside the smallest possible tube relative to sample size. This maintained a vertical orientation of plant samples for optimal rotational axis during imaging. LMP agarose effectively coated and stabilized even the most delicate or complicated samples, and provided complete physical support preventing tissue movement and drift during long scans. Higher scan magnifications and resolutions required longer scan times and our methods allowed both, which concomitantly enabled improved computational segmentation by providing higher quality raw image data. One recent example has been reported where conventional X-ray CT was used to image fresh plant tissue using molten petrolatum to stabilize small aquatic plants (Jones et al., 2020), although without contrast enhancement of samples it remains unclear if this method would be suitable for high-resolution XRM imaging.

Contrast enhancement with PTA has widespread utility due to broad staining of various plant tissues (Hayat, 1993; Staedler et al., 2013). As effective as PTA is as a contrast agent for most plant samples, it requires a lengthy incubation period—at least 14 d in our methods—to generate sufficient contrast. The application of microwave tissue processing could potentially accelerate PTA contrast enhancement protocols down to days or even hours (Kang et al., 1991; Zechmann and Zellnig, 2009; Carpentier et al., 2012). In our hands, conventional fixation in buffered aldehydes followed by post-fixation with osmium tetroxide and potassium ferrocyanide was effective in contrasting samples like root tips and Arabidopsis plantlets, but penetration was limited into larger complex floral structures. Iodine-based contrast agents can rapidly contrast entire plant tissues within 24 h, but they easily leach out of specimens into the surrounding agarose, lowering effective sample contrast and increasing scan noise and background. Alternatively, more rigid plant specimens can be contrasted in iodine and instead of agarose, stabilized with relatively X-ray transparent polyester fibers or small polystyrene beads, adding pieces of paper towel or lab wipes saturated with water or ethanol to maintain high relative humidity before sealing in sample tubes. Other robust plant materials such as wood samples (Reynolds et al., 2018) or seeds and grains (Su and Xiao, 2020; Han et al., 2021; Matsunaga and Smith, 2021) are readily imaged with XRM with a minimum of preparation. Supplemental Figure S8 shows XRM scans of maize, sorghum, soybean, and wheat seeds where no fixation or contrast enhancement were used. Seeds were simply placed in PCR or Eppendorf tubes and stabilized with expanded polystyrene beads, to observe a range of internal seed structures.

Meristematic structures, which are often too large and dense for meaningful 3D imaging using fluorescence or EM, can greatly benefit from XRM. Numerous recent studies have provided important insights into meristematic and inflorescence structure development, including *S. viridis* (Yang et al., 2018), *Hordeum vulgare* (Walla et al., 2020), maize (Teng et al., 2020), *Aquilegia coerulea* (Min and Kramer, 2020), and other grasses (Kellogg et al., 2013; AuBuchon-Elder et al., 2020). These examples illustrate the breadth of research questions that can be enhanced by additional tissue- and cell-level 3D XRM imaging to measure and evaluate meristematic structure development over scales not possible or practical with other methodologies. To address the challenge of meristematic cells having a relatively uniform density between the cytoplasm and cell walls, nuclei can be used as foci for segmentation since ePTA imparts high contrast to nucleic acids and the location of the nucleus in the cell can assist computational efforts to identify individual cells. This may require higher magnification/resolution scans to improve segmentation. Segmentation of meristematic tissues nonetheless remains challenging even with high-resolution scans of well-contrasted samples, each new sample requiring a unique combination of automated and manual segmentation solutions to accurately and reliably separate and identify specific cells and tissues. Additional computational tools for cell segmentation are emerging and should provide powerful options for the analysis of 3D volumes from a wide range of plant samples (Wolny et al., 2020).

The use of contrast agents for meristematic tissue is critical if cell-level segmentation is the goal. Thus, the development and application of cell wall-specific chemistries such as electron-dense probes or particles to generate XRM contrast agents that are specifically selective for plant and fungal cell walls would be highly beneficial. While selective staining may be possible using affinity-based small molecules (e.g. gold nanoparticles), we also envision the application of genetically encoded expression of ascorbate peroxidase-fluorescent protein fusions to a library of select plant cellular compartments (Ariotti et al., 2018). The ascorbate peroxidase method works by producing localized expression of an osmiophillic precipitate that can be contrasted by light, X-ray and electron microscopy when the ascorbate peroxidase fusion protein is incubated with 3,3-diaminobenzidine (DAB) and hydrogen peroxide (Martell et al., 2017). This would allow osmium-reactive DAB deposits at target structures that could be visualized first by XRM and subsequently processed for high-resolution EM studies as described previously (Ng et al., 2016; Tsang et al., 2018; Kong et al., 2020).

Plant–microbe interactions, root biology, and vascular bundles were readily differentiated by XRM imaging with relatively straightforward sample preparation. For example, root nodules induced on soybean were visualized in detail (Figure 4, A–D) without the time consuming and technically challenging tasks of physical embedding and sectioning, acquiring 2D photomicrographs of each section, and manually assembling serially organized images into a 3D model to study root nodule morphology and vasculature (Livingston et al., 2019). Mycorrhizal fungi that form arbuscules within cortical cells of plant roots for mutualistic nutrient exchange between plant and fungus can be visualized and quantified as shown by segmentation of *R. irregularis* in maize roots (Figure 4, E and F). We also developed a syringe barrel system where both host root system architecture and fungal symbiont were imaged *in situ* (Figure 4, G and H; Supplemental Figure S7) without perturbation of the local environment. We are currently exploring various physical and chemical affinity probes and contrast agents that could be applied directly to the growth medium for improved XRM visualization of root–microbe interactions, adapted from Scotson et al. (2019) who used X-ray CT and SEM to evaluate gold nanoparticles as a contrast agent in soil, studying the interaction of particles from a liquid suspension drawn through a column containing various media.

Cell-level measurement of roots in 3D is now possible with XRM imaging to better understand how simple root growth responses such as elongation, bending, and branching result in complicated and vast root networks that occupy square meters of soil (Figure 3, A–D; Supplemental Figures S5 and S6). This enables studies that compare cell number, morphology, and 3D position in root tips between maize lines known to differ in root system architecture at relevant developmental stages (Jiang et al., 2019), or for modeling hydraulic conductivity (Heymans et al., 2020). Iodine-based contrast agents were useful for visualization of vascular elements (Figure 3E), and coupled with XRM will be of great value for mapping physically complex regions such as the root–shoot transition zone (nodal plexus) and graft junctions between rootstock and scion. We are currently involved in multiple collaborations using XRM to image and evaluate specific genetic effects on morphology and development of samples across a range of plant species, which we anticipate will further demonstrate specific uses of XRM for generating biologically meaningful insights.

Imaging of EM prepared resin-embedded specimens in the life sciences increasingly has been applied to 3D XRM (Hanssen et al., 2012). Likewise, protocol improvements in SBFEM sample preparation techniques (Wanner et al., 2015; Titze and Genoud, 2016) including the protocol used here with *en bloc* metal staining (Hua et al., 2015), demonstrated improved osmium penetration and enhanced contrast in thick plant tissues which inherently benefit high-resolution XRM and SBFEM correlative workflows (Bushong et al., 2015). While there have been a handful of published plant-based SBFEM examples (Kittelmann et al., 2016; Oi et al., 2017; Czymmek et al., 2020; Harwood et al., 2020), we reasoned that further improvements in uniformity, conductivity and intensity of plant staining (Deerinck et al., 2018) would substantially benefit both XRM and SBFEM image contrast and quality for correlative studies. Additionally, specimens encapsulated in rigid hard formulation epoxy resins provided excellent stability for long-duration XRM scans, translating to the optimal resolution. Our protocol resulted in optically opaque specimens in resin blocks that initially were scanned with XRM to identify regions of interest and guided our subsequent high-resolution volume EM, all from the same block and using a single optimized sample preparation method. Notably, we believe these data reflect the highest resolution reconstruction of subcellular plant structures via microCT or XRM published to date, allowing sub-organellar starch segmentation via deep learning (Figure 5G). We envision that our plant-based correlative XRM to EM workflow will serve as an effective way to obtain 3D contextual histology, as a standalone approach, or modified to include other correlative strategies such as targeting genetically encoded structures and/or combined with light microscopy approaches. While less accessible, we acknowledge that further resolution gains using synchrotron-based platforms will allow nanoscale imaging (Hwu et al., 2017; Fonseca et al., 2018; Chin et al., 2020) also benefiting from ours and others’ plant sample preparation strategies. Ultimately, these tools and protocols will accelerate the ease in localizing, resolving, and quantifying important discrete and multiscale plant biological phenomena, including developmental studies, phenotype characterization, and plant–microbe interactions, for subsequent high-resolution EM studies.

## Conclusion

We have demonstrated that lab-based XRM provides a unique means of 3D imaging larger and more complex plant samples than is typically possible for light and electron microscopy, providing researchers with an opportunity to visualize important features in high resolution over a wide range of magnifications within the context of larger tissues, organs, or whole plants. The use of XRM to augment volume electron microscopy by providing a high-resolution 3D roadmap makes multiscale correlative imaging a powerful option for visualization of sub-cellular and cellular details, again co-registered across scales and within a larger sample context. We hope that describing numerous examples of robust, high-resolution 3D imaging strategies from a wide range of plant samples will encourage more researchers to explore lab-based XRM as a powerful and essential tool for understanding cell, tissue, and organismal plant biology, as well as assist technical staff in imaging facilities who may not have experience in XRM imaging of plant samples.

## Materials and methods

### Sample contrast and mounting

A preparation overview for specific samples is provided in Supplemental Table S1, as well as a workflow diagram illustrating how samples are fixed, stabilized, and mounted for XRM imaging in Supplemental Figure S1. General methods and correlative microscopy details are described in the following section. Most samples were fixed directly in 20-mL glass vials in the contrast agent, ePTA, using 1%PTA (w/v) in 90% ethanol in ddH_2_O (v/v). A brief (∼5 min) vacuum was applied to facilitate penetration of ePTA, particularly for hydrophobic plant tissue and floral structures with prolific trichomes, especially leaves. Samples remained in ePTA between 14 and 56 d on a mechanical lab rocker, with exchanges of ePTA every 7 d. While incubation in ePTA <14 d gave inferior contrast and penetration, prolonged incubation did not adversely affect imaging and in most cases enhanced contrast. Some samples were fixed in 4% glutaraldehyde and 4% paraformaldehyde in 1× phosphate-buffered saline (PBS) at pH 7.4. Samples were exposed to several cycles of vacuum/air until samples sunk after vacuum release. After aldehyde fixation overnight at 4°C, samples were rinsed 3 × 30 min in PBS, then post-fixed in 1% osmium tetroxide (v/v) with 1% potassium ferrocyanide (v/v) in PBS for 24 h at room temperature. Post-fixed samples were rinsed 3 × 60 min in PBS then stored at 4°C.

Fixed and contrast-enhanced samples were rinsed 10 min in ddH_2_O on a lab rocker. LMP agarose (Promega #V2111) was prepared at 1% (w/v) in ddH_2_O on a laboratory hot plate and removed from heat just as the agarose melted, before active boiling. A narrow transfer pipette was used to add enough agarose to the bottom of a 200 µm flat cap PCR tube to ensure the sample would be completely submerged when transferred. Fine forceps were used to transfer the sample from the ddH_2_O rinse vial, blotting briefly on a paper towel to remove excess ddH_2_O, then placed in the liquid agarose in the base of the tube. Additional agarose was added to completely fill the tube, avoiding air spaces or bubbles. After allowing agarose to set, the cap was closed tightly and a two-component epoxy gel (Devcon, ITW) was used to completely seal the tube and to mount the tube on the end of a wooden applicator stick. Epoxy was allowed to harden for at least 60 min before loading into the XRM sample holder (process illustrated in Supplemental Figure S1).

LMP agarose is our preferred medium for stabilizing small and delicate samples, particularly for long high-resolution scans. In some cases a small plug of polyester fibers is added to the molten agarose over the sample for additional stability, as long duration high-resolution scans can sometimes liquefy the agarose immediately surrounding the sample. If samples are larger and more robust, they can be stabilized in a variety of low-density media, which then allow the use of iodine-based contrast agents such as Lugol’s iodine-potassium iodide solution. Lugol’s was prepared as 500 mL of a 4× stock solution by adding 50 g of potassium iodide to 400 mL ddH_2_O and stirring until dissolved, followed by adding 25 g iodine and adding ddH_2_O to a total volume of 500 mL. Polyester fibers typically used for pillows can be used to pack and cushion larger samples in appropriately sized plastic tubes. The fibers can be carefully moistened with ethanol or water—depending on whether the contrast agent was ethanolic or aqueous—before sealing the tube to ensure a stable relative humidity over the course of the scan. Similarly, cotton wool can be used although the cotton fibers also absorb iodine (Lugol’s) which can complicate subsequent image segmentation. Finally, small expanded polystyrene beads in the 1–2 mm range, available online or at most hobby/craft shops, can be used to stabilize larger samples like seeds, grains, and maize stalk segments.

### Correlative microscopy

*Nicotiana* *benthamiana* leaves were fixed, stained, and embedded using a modified protocol for enhanced *en bloc* staining of large tissues (Hua et al., 2015) that was compatible for high-resolution XRM and SBFEM imaging. Briefly, 3-mm biopsy punches of leaves were fixed overnight with 2% glutaraldehyde (v/v) and 2% paraformaldehyde (v/v) with 0.1% Tween (v/v) in 0.1 M sodium cacodylate buffer, pH 7.4, rinsed in 3× in buffer and fixed for 2 h in 1% OsO_4_ (v/v) in cacodylate buffer at room temperature. Samples were then transferred to 2.5% ferrocyanide (v/v) in cacodylate buffer for 1.5 h, rinsed and placed in 1% thiocarbohydrazide (w/v; TCH, #21900; Electron Microscopy Sciences) in ddH_2_O for 45 min at 40°C. Subsequently, samples were then fixed with 2% OsO_4_ (v/v) in ddH_2_O for 1.5 h, rinsed 2× in ddH_2_O, and placed overnight in aqueous 1% uranyl acetate (w/v) at 4°C. The samples were then transferred to an oven at 50°C for 2 h, rinsed, placed in lead aspartate at 50°C for 2 h, rinsed, dehydrated in a graded series of acetone, and embedded in Embed 812 hard formulation epoxy resin.

Resin samples were trimmed, mounted with epoxy glue on an aluminum pin and imaged on a Zeiss Xradia 520 Versa using a 4× objective (Supplemental Table S2). For 40× imaging, a Zeiss Xradia 620 Versa was used, pixel size was 0.19-µm isotropic, exposure time is 27 s, and 3001 projections were taken over 360 degrees. No filter was used with a 70-kV and 8.5 W power. Following scans, Optirecon 2.0 (Advanced Reconstruction Toolbox) deconvolution processing was applied. For SBFEM, the sample was then remounted on a Gatan 3View aluminum pin with 2-part conductive silver epoxy (Circuit Works, CW2400), trimmed to minimize excess resin and sputter coated with AuPd before sectioning on a Zeiss GeminiSEM300 equipped with a Gatan 3View at 1.2 kV with focal charge compensation (Deerinck et al., 2018) using 8,000 × 8,000 pixel image size and 5-nm pixel size (*x*–*y*) and 50 nm slice thickness (*z*).

### X-ray microscopy

Apart from the 40X scan described above, all scans were conducted on a Zeiss Xradia 520 Versa, equipped with four objective lenses (0.4X,4X,20X,40X), as well as a 3072 x 553 1944 pixel flat panel detector with 75 sm pixel pitch. Scan parameters for all scans are 554 listed in Supplemental Table S2. Zeiss Reconstructor software was used to automatically or manually reconstruct 3D volumes from 2D scan data, and Object Research Systems (ORS) DragonflyPro software (Montreal, Canada) was used for data integration, visualization, and animation of the scan data, and to export image data as 2D 16-bit Tag Image File Format (TIFF) stacks. Fly-through animations of 2D image stacks for scans shown in all Figures, as well as 3D volume rendering animations of selected scans, are available for download from (https://figshare.com/s/944efc8832e47fd4f203).

### Image analysis and segmentation

Data from XRM scans were segmented using Amira software and with the assistance of a Wacom tablet for manual segmentation, in addition to ORS Dragonfly Deep Learning Module 2021.1.0.977 which is free for noncommercial use. Segmentation for Figure 1D combined automated and manual methods in Dragonfly. The Dragonfly Segmentation Wizard was used to train a Sensor3D model using seven manually segmented three-phase training frames to identify spikelets, bristles/panicle, and air based on grayscale and on object shape. The trained model was then used to segment the inflorescence structure scan volume. The segmented volume was then adjusted manually with Dragonfly to distinguish regions of spikelets and bristles that were not correctly identified by the model. Segmentation for Figure 3B used automatic and manual methods as follows: (1) Based on thresholding, the mask image of root tip was created; (2) H-maxima transform was applied to the mask image; (3) watershed segmentation was applied to segment individual cells; (4) different cell classes were labeled manually. Segmentation for Figure 3C used semi-automatic method as follows: (1) different thresholding values were used to segment different layers; (2) morphological operations (opening and closing) were applied to smooth the boundaries of layers; (3) boundaries were manually corrected or drawn through the cross-section images. The individual cells of the soy nodule in Figure 4, C and D were segmented using marker-based watershed segmentation as follows: (1) symbiosomes were segmented using a threshold value; (2) nuclei were segmented using a higher threshold value; and (3) a marker-based watershed method with nuclei as markers, was applied to segment individual cells. The vasculature was manually segmented from a TIFF stack of the volume from Figure 4B using Amira and a Wacom tablet and then combined with an automatic segmentation using region growing and morphological closing operation. Segmentation for Figure 4F used automatic and manual methods as follows: (1) based on thresholding, the mask image of the root was created; (2) H-maxima transform was applied to the mask image; (3) watershed segmentation was applied to segment individual cells; (4) Arbuscules were segmented using thresholding and component filtering. For Figure 4H, spores were manually segmented from the TIFF stack using Amira and a Wacom tablet. The original grayscale volume was then cropped along the *Z*-axis to show the locations of the spores throughout the volume. For correlative XRM data (Figure 5F), we used ORS Dragonfly Deep Learning Module with the Sensor3D model (Novikov et al., 2019), which takes three slices into account for each slice segmented with Dice loss and AdaDelta optimization method for training. We then used a 128 × 128 pixel image patch with a stride ratio of 0.5 and a slice step of 2 (the slice 2 below and 2 above are used as input along with the current slice). Twenty slices were trained and data augmentation set to a factor of 15, with rotation, horizontal and vertical flipping, shearing (of 2 degrees), and scaling (0.9 to 1.1).

## Supplemental data

The following materials are available in the online version of this article.

**Supplemental Figure S1**. Sample preparation workflow for XRM.

**Supplemental Figure S2**. Tissue level segmentation of *Setaria viridis* spikelets and bristles.

**Supplemental Figure S3**. Inflorescence development.

**Supplemental Figure S4**. Multiscale imaging.

**Supplemental Figure S5**. Identification and 3D volume measurement of individual cells from XRM scan of maize root tip.

**Supplemental Figure S6**. Measurement data from 3D segmentation of maize root tip XRM scan.

**Supplemental Figure S7**. Multiscale *in situ* imaging of host–microbe interaction.

**Supplemental Figure S8**. Seed morphology.

S**upplemental Table S1**. Preparation details for all samples illustrated in figures.

**Supplemental Table S2**. X-ray microscope scan parameters for images shown in figures.

## Supplementary Material

kiab405_Supplementary_Data

## Footnotes

1 high-resolution image stacks (“flythroughs”) and videos portraying the 3D data sets can be found here: https://figshare.com/s/944efc8832e47fd4f203

## Notes

K.E.D., K.J.C., and C.N.T. designed the research; K.E.D. and K.J.C. performed the research; N.J., A.C.T., K.J.C., and K.E.D. provided computational analyses; K.E.D., K.J.C., and C.N.T. wrote the article.

The author responsible for distribution of materials integral to the findings presented in this article in accordance with the policy described in the Instructions for Authors (https://academic.oup.com/plphys/pages/General-Instructions) is: Christopher N. Topp (CTopp@DanforthCenter.org).

## Notes

*Conflict* *of interest statement*. None declared.
