# Supplementary material for: X-ray microscopy enables multiscale high-resolution 3D imaging of plant cells, tissues, and organs
Source: Plant Physiol. 2021 Sep 27;188(2):831–45. doi: 10.1093/plphys/kiab405 (PMC8825331; doi:10.1093/plphys/kiab405)
Supplement: kiab405_Supplementary_Data [file kiab405_supplementary_data.zip › SupplementaryFigures_Rev3.pdf]

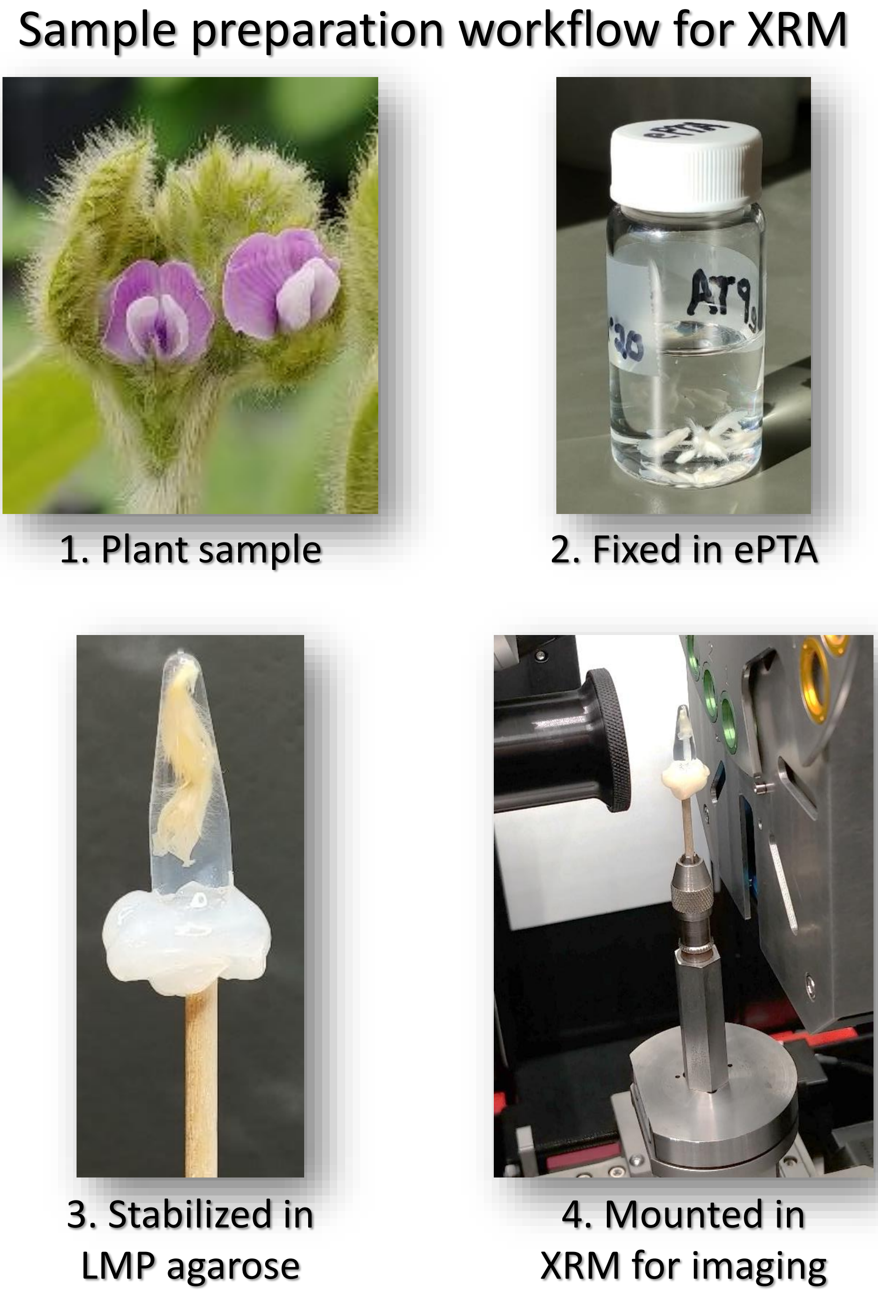

Supplemental Figure S1. Live samples are removed from the plant (**1**), fixed and contrast enhanced in ethanolic phosphotungstic acid (ePTA,**2**), stabilized in PCR or centrifuge tubes using low melting point (LMP) agarose and affixed to a wooden applicator stick with two-cycle epoxy gel (**3**), then mounted on XRM sample holder for imaging (**4**).

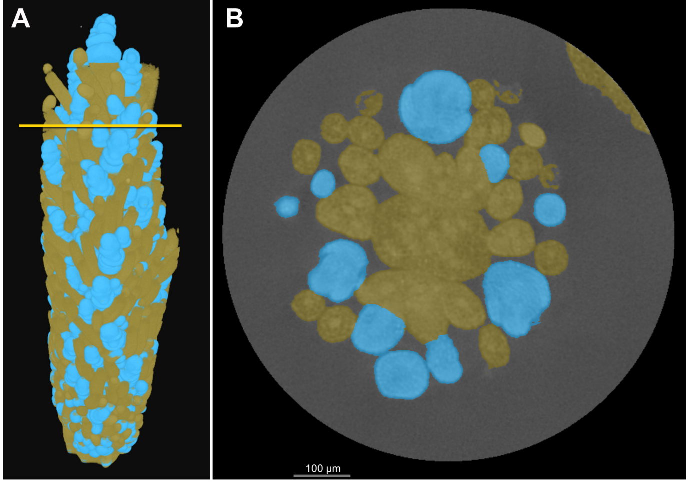

Supplemental Figure S2. Tissue level segmentation of *Setaria viridis* spikelets and bristles

A Computational segmentation of *S. viridis* inflorescence structure from **Fig 1D**. A combination of ORS Dragonfly Deep Learning Segmentation and manual curation were used to separate the scan volume into spikelets (blue) and sterile bristles and panicle (tan). 3D volume segmentation was used to calculate the volume of spikelets (20.3%) relative to the total inflorescence structure (79.7%), a useful measure when comparing scans of different inflorescence samples. **B** 2D slice through 3D volume segmentation at region indicated with yellow line.

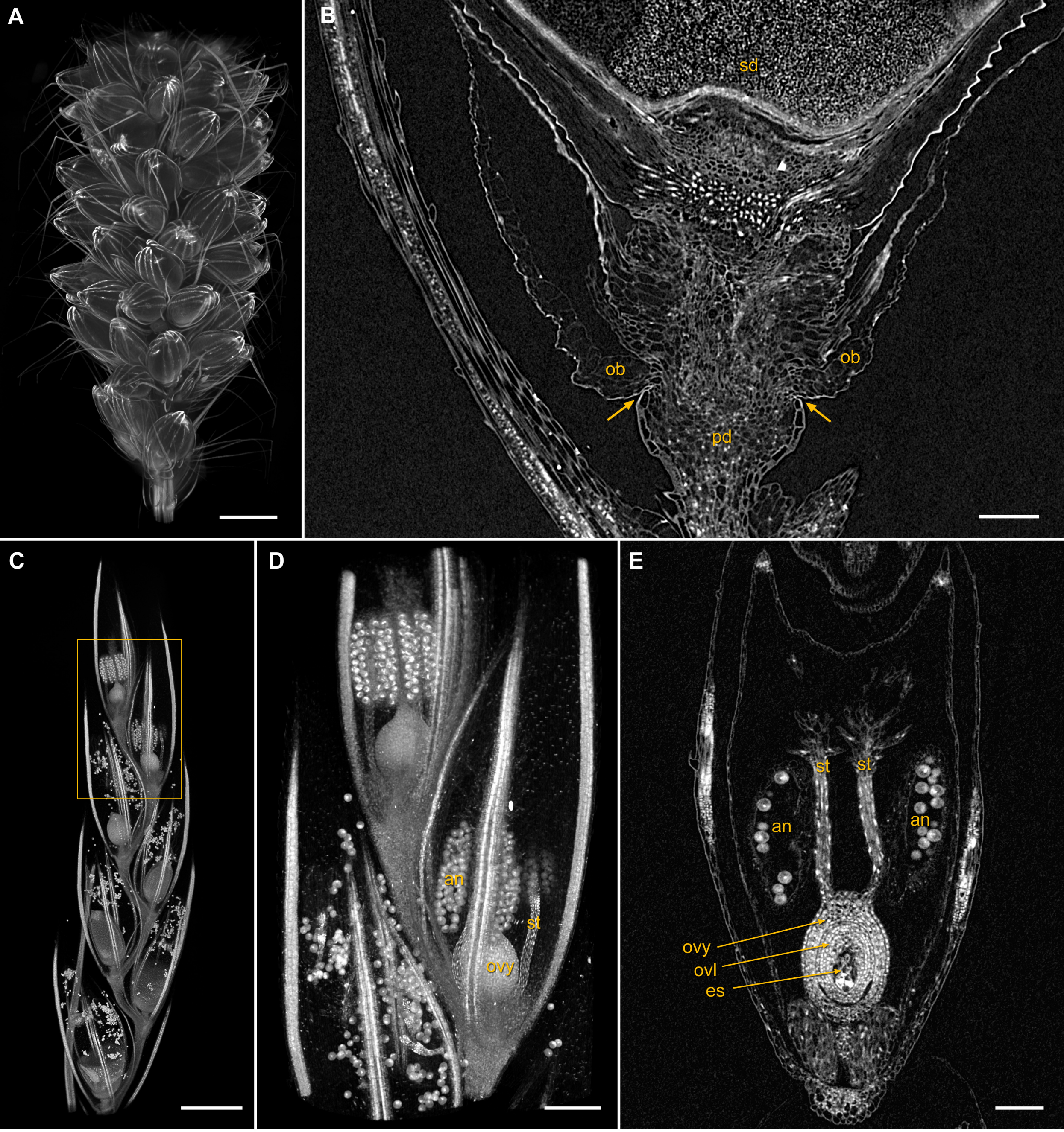

Supplemental Figure S3 Inflorescence development

A 3D volume rendering of a *Setaria viridis* inflorescence with seed-containing spikelets and sterile bristles visible; scale bar 3 mm. **B** High resolution image of a single spikelet from **A**, arrows indicate the region of the outer bracts (**ob**) and pedicel (**pd**) where the abscission zone will form as the seed (**sd**) matures; scale bar 100 um. **C** 3D volume rendering of inflorescence structure from *Eragrostis tef*; scale bar 1 mm. **D,E** High resolution scan of indicated region from **C**. Structures such as the ovary (**ovy**), anthers (**an**), and stigmas (**st**) are readily distinguished in the 3D volume rendering (**D**), with the ovule (**ovl**) and embryo sac (**es**) visible in the 2D clip plane (**E**); scale bars 200 um (**D**), 100 um (**E**),

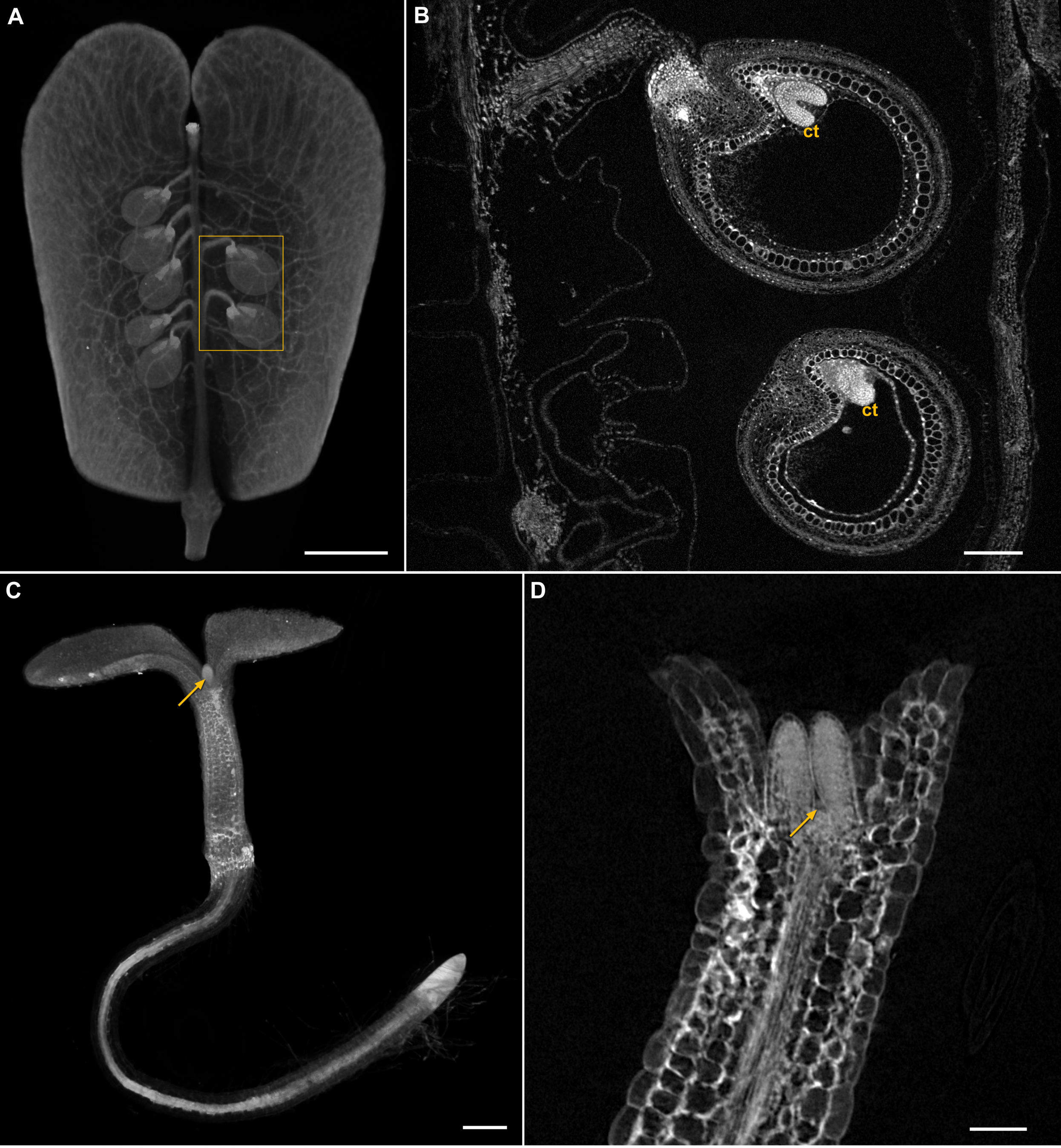

Supplemental Figure S4. Multiscale imaging

Examples of how the same sample can be imaged over multiple scales without removing it from the instrument. **A,B** Single pod at seed-filling stage of pennycress (*Thlaspi arvense*), fixed and contrast enhanced, stabilized in agarose, and imaged at low (**A**) and high (**B**) resolution. Cotyledons (**ct**) and multiple seed layers are clearly visualized; scale bars 1.5 mm (**A**), 200 um (**B**). Arabidopsis seedling fixed and contrast enhanced, stabilized in agarose, and imaged at low (**C**) and high (**D**) resolution. Arrows point to the developing shoot apical meristem; scale bars 200 um (**C**), 50 um (**D**).

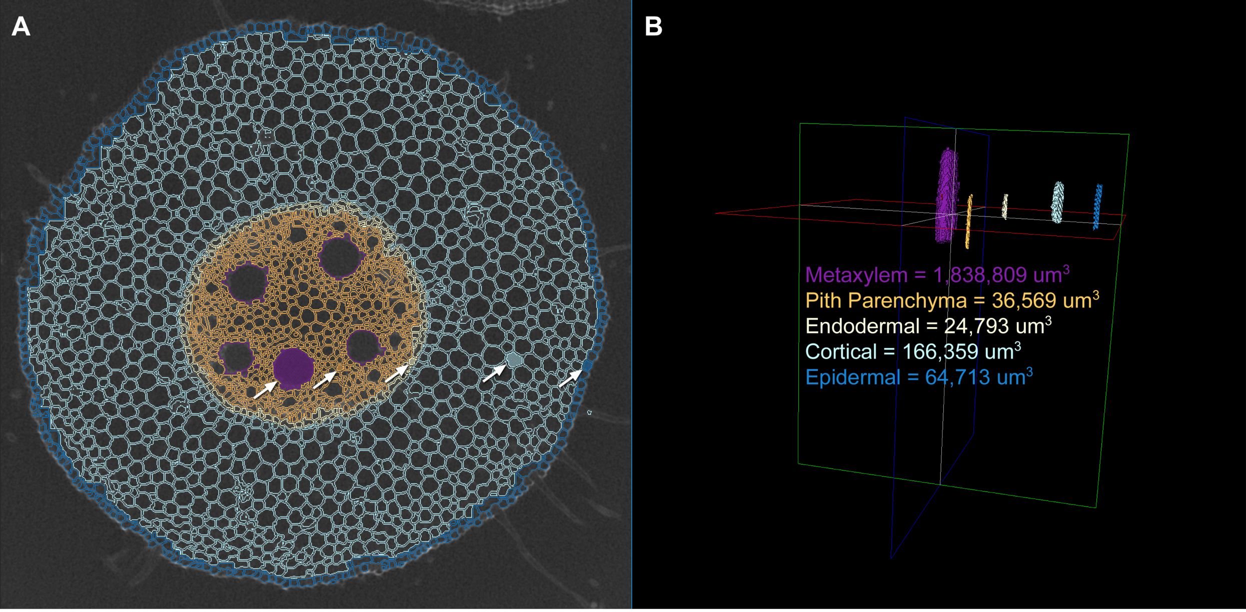

Supplemental Figure S5. Identification and 3D volume measurement of individual cells from XRM scan of maize root tip

A Segmented 2D slice of maize root tip from **Fig 3B** with single shaded cells selected for 3D volume measurement (**arrows**). **B** 3D volume display and measurements of five individual cells from **A**: epidermal (blue), cortical (cyan), endodermal (beige), pith parenchyma (orange), and metaxylem (purple).

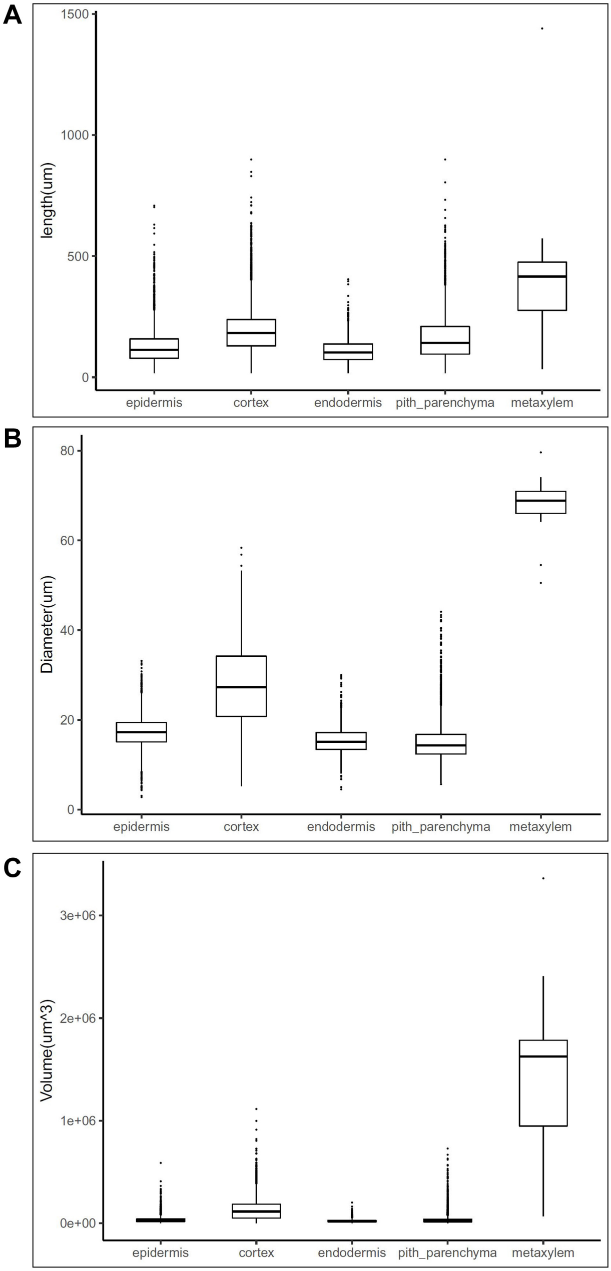

Supplemental Figure S6. Measurement data from 3D segmentation of maize root tip XRM scan

A-C Boxplots of 3D measurements based on segmentation data from XRM scan of maize root tip shown in **Figs 3B,C** and **Supplemental Fig S5**, showing individual cell length (**A**), diameter (**B**), and volume (**C**). Boxplots were generated in ggplot using geom_boxplot function. In the boxplots, the center line is the median, the lower and upper hinges correspond to the first and third quartiles. The upper whisker extends from the hinge to the largest value no further than 1.5 x IQR from the hinge. The lower whisker extends from the hinge to the smallest value at most 1.5 x IQR of the hinge. Data beyond the end of the whiskers are outlying points and are plotted individually.

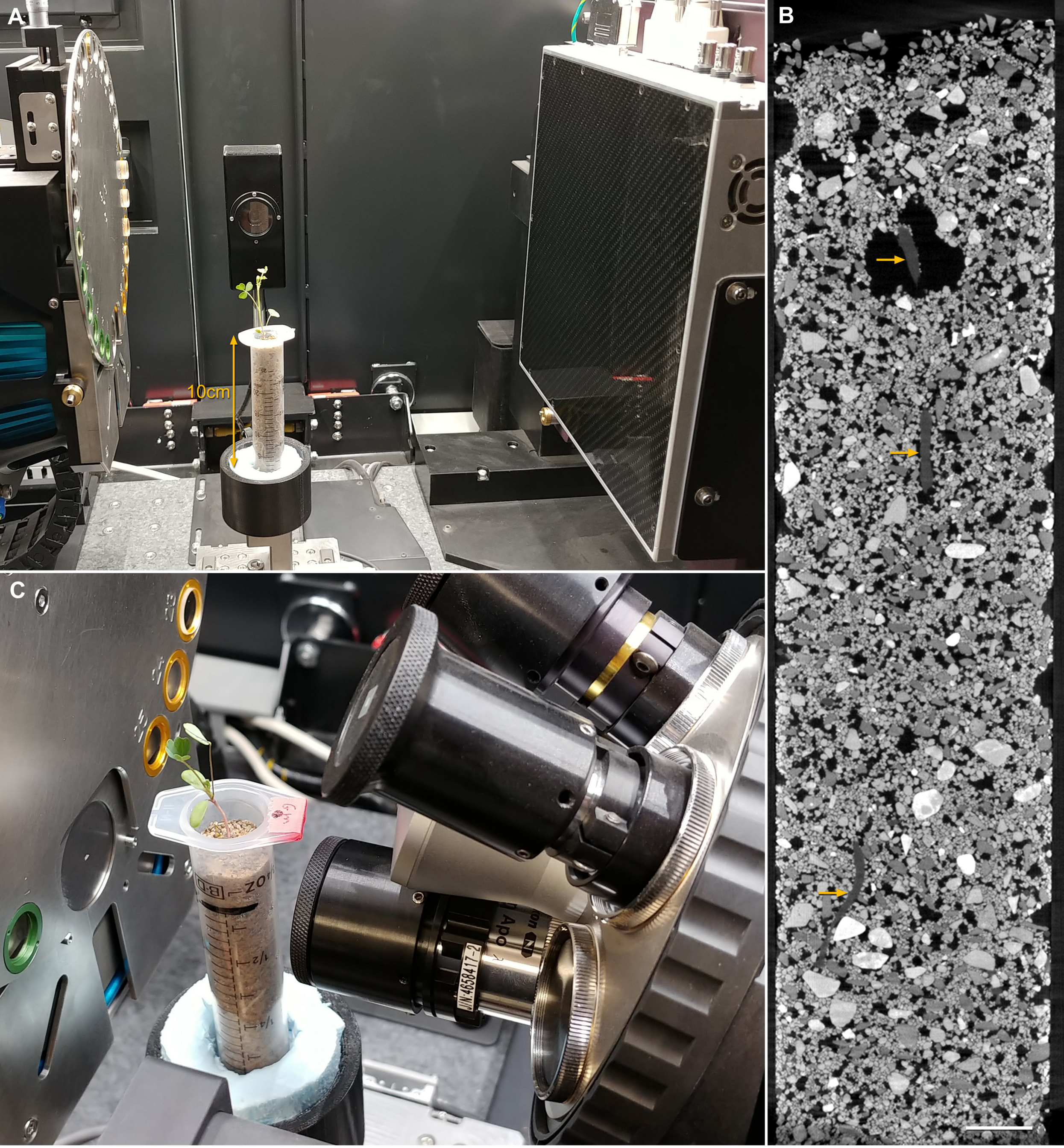

Supplemental Figure S7. Multiscale *in situ* imaging of host-microbe interaction

Medicago sativa and mycorrhizal fungus *Gigaspora margarita* interaction visualized over a wide range of magnifications. Imaging with a flat panel detector (**A**) allows visualization of *M. sativa* root system architecture in the entire 2 cm x 10 cm syringe barrel volume (**B, arrows**); scale bar 5 mm. Image data from **B** directs high resolution 3D imaging with a 4X objective lens (**C**), where segmentation of individual *G. margarita* spores is presented in **Fig 4G,H**.

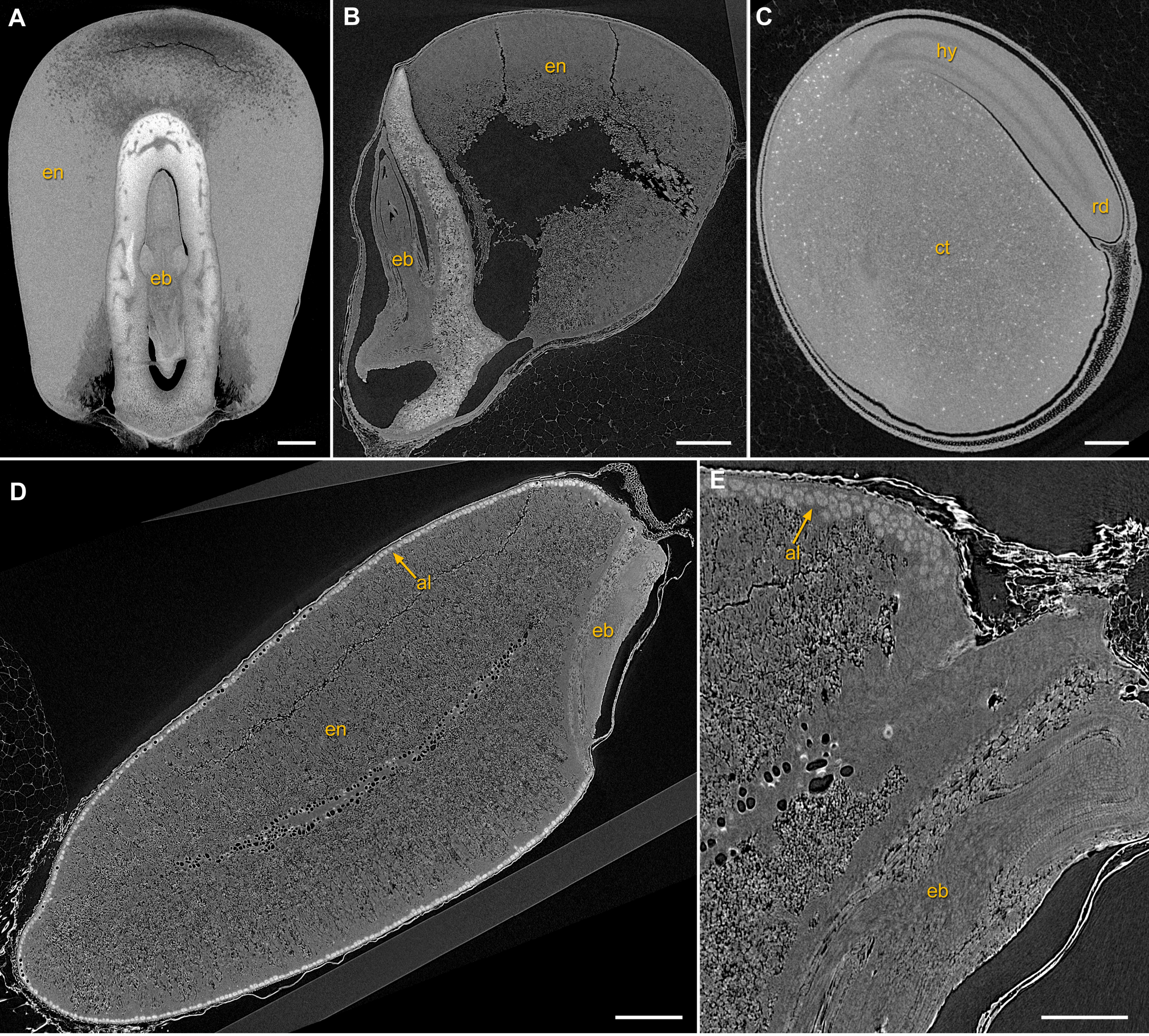

Supplemental Figure S8. Seed morphology

Seeds from maize (**A**), sorghum (**B**), soybean (**C**), and wheat (**D,E**) were scanned without fixation or contrast enhancement. Samples were stabilized in PCR or centrifuge tubes packed with expanded polystyrene beads. Internal structures such as endosperm (**en**), embryo (**eb**), cotyledon (**ct**), hypocotyl (**hy**), radicle (**rd**), and aleurone layer (**al**) are readily visualized; scale bars 1 mm (**A**), 500 um (**B,C,D**), 200 um (**E**).
